# Toxicological Vulnerabilities and Emerging Safety Signals in Modified mRNA Vaccine Technology: A Narrative Review of the COVID‐19 Experience

**DOI:** 10.1155/jt/8202757

**Published:** 2026-10-15

**Authors:** Gerry A. Quinn, Philipp Bagus, Antonio Sánchez-Bayón, Ronan Connolly, Clare Craig, Paul Frijters, Steven J. Hatfill, Anton O’Connor, Daniel Santiago, Ricardo F. Savaris, Michaéla C. Schippers, Willie Soon, Demetrios A. Spandidos, Helmut Sterz, Aristidis M. Tsatsakis, Harald Walach

**Affiliations:** ^1^ Centre for Molecular Biosciences Ulster University Coleraine BT521SA UK ulster.ac.uk; ^2^ Center for Environmental Research and Earth Sciences (CERES) Salem Massachusetts USA; ^3^ Department of Applied Economics Faculty of Law and Social Sciences Rey Juan Carlos University Móstoles Madrid 28933 Spain urjc.es; ^4^ Department of Applied Economics Rey Juan Carlos University (URJC & CINTTEC-FJHSB, ref. V1735) Móstoles 28933 Spain urjc.es; ^5^ Independent Scientist Dublin D08 Ireland; ^6^ Health Advisory and Recovery Team London UK; ^7^ Department of Social Policy London School of Economics London WC2A 2AE UK lse.ac.uk; ^8^ London Center for Policy Research New York 10016 New York USA; ^9^ Independent Pharmacist Orlando 32807 Florida USA; ^10^ Department of Gynecology and Obstetrics Faculty of Medicine Federal University of Rio Grande do Sul Porto Alegre 90035-003 Brazil ufrgs.br; ^11^ Rotterdam School of Management (RSM) Erasmus University Rotterdam Rotterdam 3062 PA Netherlands eur.nl; ^12^ HUN-REN Institute of Earth Physics and Space Science Sopron 9400 Hungary; ^13^ Laboratory of Clinical Virology Medical School University of Crete Heraklion 71003 Greece uoc.gr; ^14^ Independent Researcher Tours 37100 France; ^15^ Center of Toxicology & Science Applications Medical School University of Crete Heraklion 71003 Greece uoc.gr; ^16^ Human Development and Health Science Faculty Universidad ECOTEC km. 13.5 Samborondón Samborondón EC092302 Ecuador ecotec.edu.ec; ^17^ Biomedical Science and Technology Park Sechenov I.M. First State Medical University Moscow 119991 Russia; ^18^ Next Society Institute Kazimieras Simonavičius University Vilnius LT-02189 Lithuania

**Keywords:** biodistribution and persistence, frameshifting, lipid nanoparticles, modRNA vaccine platform, nucleoside modification, postauthorisation observations, risk–benefit assessment, safety and adverse events

## Abstract

The rapid deployment in early 2021 of modified mRNA (modRNA) as a vaccine platform for the COVID‐19 pandemic sparked a wave of optimism for its potential as a preventive medicine. However, this compacted timeframe may not have fully addressed certain toxicological risks associated with this new technology, which would typically require more extensive preclinical testing. Five years postrollout, an accumulating body of postauthorisation evidence has highlighted safety signals that require closer scrutiny, such as those concerning biodistribution, off‐target effects and persistent adverse events. Independent analyses of Phase III trial data and pharmacovigilance reports have raised questions regarding benefit–risk balance and potential long‐term biological effects. This narrative review examines the evolution of the toxicological understanding of modified RNA as a vaccine technology from platform design through long‐term observational data, identifying areas of uncertainty and vulnerability that may inform future development and regulatory evaluation. The review emphasises that fast‐tracked decision‐making under exceptional uncertainty requires explicit safeguards to prevent premature certainty and to support learning from emerging evidence over time.

## 1. Introduction

Unlike storytellers, who craft narratives with predetermined outcomes, scientists in public health and toxicology must rely on empirical data to predict risk. With the emergence of new genetic‐based technologies, this consideration demands extra vigilance, as unforeseen risks can only emerge after widespread use of a product, potentially leading to irreversible harm. To compound these limitations, periods of emergency, such as the COVID‐19 pandemic which started in 2021, place unusual constraints on scientific assessment, including compressed timelines, heightened expectations of decisive action, and limited opportunities for systematic reassessment. In such settings, without explicit evaluative safeguards, early interpretations risk becoming entrenched before sufficient evidence accumulates [1, 2].

In early 2021, many health authorities around the world simultaneously rolled‐out a new technology that they described as a COVID‐19 vaccine. This was a revolutionary departure from traditional vaccines, which normally use the whole or part of a pathogen in an attenuated or inactivated form to stimulate an immune response. Instead, this new technology involved introducing the genetic code for a pathogenic viral protein, encapsulated in a lipid nanoparticle (LNP), which cells would then translate into protein. It was further theorised that the immune system would then mount an appropriate immune response against this foreign protein.

### 1.1. The Development of mRNA/Modified RNA (modRNA) Vaccine Technology

The core technologies underlying the genetic‐based vaccines that were deployed against COVID‐19 have been under consideration for decades. However, the major barrier to their introduction has always been their extreme instability and the strong innate immunogenicity of naked mRNA. In 1989, Robert Malone and colleagues effectively solved some of these technical issues when they demonstrated that mRNA could transfect mammalian cells and direct protein expression (including influenza hemagglutinin) when delivered via the protective envelope of cationic liposomes [3]. However, there still remained the problem of the immune detection of foreign RNA. This problem was addressed by Katalin Karikó and Drew Weissman, who found that replacing uridine with N1‐methylpseudouridine (m1Ψ) dramatically reduced the host immune activation potential while increasing RNA stability and translation efficiency. This technology became known as modRNA [4, 5]. Subsequent development of specialised ionisable LNPs, which were initially developed by Robert Malone, provided protection against modRNA degradation, enabled efficient cellular entry, and further masked the modRNA from early immune detection [6].

While these advances addressed key technical barriers to RNA delivery and stability, they also altered fundamental interactions between synthetic nucleic acids and host biology. At the time of deployment, several aspects of these interactions, including biodistribution, persistence, and downstream cellular effects, were incompletely characterised, raising questions that have since become the subject of increasing scientific scrutiny.

Prior to the COVID‐19 vaccine trials, only 285 individuals had received mRNA vaccines, all within recent clinical trial settings [7]. Therefore, given the widespread use of this technology worldwide, side‐effect profiles are still being established. Given the hypothetical mechanisms that might lead to harm, some of these cases might lead to systemic multiorgan effects rather than specific rare conditions.

### 1.2. The Mechanism of Action of modRNA Vaccines

It was assumed by developers of the modRNA vaccine that once it was administered (by intramuscular injection), the LNPs (that contain the gene sequence) would fuse with human cells and releases the modRNA into the host cell cytoplasm [6]. It was further assumed that the modified viral spike protein genetic code would then be translated by the cells’ protein synthesis machinery (ribosomes) into exact three‐dimensional replicas of the SARS‐CoV‐2 spike protein. Once this was completed, it was believed the viral spike protein would be transported via the endoplasmic reticulum/Golgi pathway to the cell surface and displayed on the outside of the cell. In theory, circulating immune cells should recognise the foreign viral spike protein as nonself and trigger an appropriate humoral (antibody) and cellular immune response. It has to be noted that this pathway is not cell‐specific and therefore runs the risk that cell entry of modRNA would lead to cell death regardless of what cell type is affected. In this regard, LNPs are an integral component of this process, as they enable efficient modRNA delivery; consequently, the biological effects of LNPs are significantly intertwined with those of the modRNA payload and should not be isolated from the overall vaccine product data. Recent reports highlight the inherent complexity and unpredictability of LNP behaviour in vivo, including batch‐to‐batch variability and long‐term stability challenges [8–11].

### 1.3. The Choice of modRNA as a Vaccine Platform for COVID‐19

The theoretical advantages proposed by modRNA include rapid design and scalable production without requiring live virus. Still, there were formidable challenges such as immune overreaction (cytokine storms), nonspecific cellular targeting, unknown antigen dose and persistence and the potential of spike proteins multiplying unchecked; issues that could potentially cascade into health crises for vaccinated populations everywhere [12–14].

At the beginning of the coronavirus pandemic, some medical and political experts likened the threat of COVID‐19 to a deadly pandemic such as Ebola. However, Ebola causes regionally heterogenous case fatality rates of approximately 25%‐60% [15], while it is now estimated that the COVID‐19 fatality rate is much less than 1% [16]. The ethical permissibility of overriding vaccine manufacturers’ established safety protocols and permitting market approval without standard toxicity studies does not depend on whether authorities anticipated Ebola‐like mortality or substantially lower fatality rates.

Given the potential risks posed by a radical departure from conventional approaches to vaccines and the experimental nature of this technology, some people have asked whether there was enough solid evidence to justify its rollout to the global population [16–18]. We briefly discussed this topic in a previous manuscript ‘What Lessons can Be Learned From the Management of the COVID‐19 Pandemic?’ [16], emphasising the unprecedented novelty and safety profile of this technology as well as other related DNA based vaccines. Since then (May 2025), the pace of research output in this area has been so fast that we have had to update some of our previous considerations on the key toxicological concerns of modRNA vaccine technology; see, e.g., Quinn et al. [19]. Therefore, the following sections evaluate the key potential risks of the modRNA platform, from its foundational design through preclinical assessments and postauthorisation observations, highlighting vulnerabilities that warrant greater scrutiny for future applications.

The subsequent sections address these concerns in detail.

## 2. Methods

Literature on the modified RNA platform was reviewed using snowball sampling (forward and backward) via Google Scholar and PubMed, using the keywords ‘risk assessment,’ ‘toxicity,’ ‘adverse events’ ‘COVID vaccine,’ and ‘vaccine injury.’

Grok, Version 3 was used to check for grammatical errors and offer suggestions for corrections.

## 3. Risk Assessment of the modRNA Vaccine Platform

### 3.1. Potential Risks of the modRNA Vaccine Platform

We have already discussed the solutions that were proposed to improve the mRNA platform including the replacement of uridine with m1Ψ and later codon‐optimisation and encapsulation in ionisable LNPs. We will now discuss potential sources of risk which stem mainly from design, manufacturing, and biology.

#### 3.1.1. Problem 1: m1Ψ Substitution: Benefits and Emerging Risks

Previous research found that the substitution of uridine by m1Ψ made the modRNA sequence less immunologically reactive to innate immune systems and markedly increased its translation [4]. While m1Ψ is a naturally occurring modification found in certain RNAs, such as archaeal transfer RNAs (tRNAs) and, in limited amounts, eukaryotic rRNAs like 18S rRNA in yeast [20] and in cancerous human HeLa cells, it is neither common nor abundant in human mRNA. In nature, for instance, archaeal tRNAs contain only 1–2 m1Ψ residues per molecule. In the Pfizer and Moderna vaccines, uridine is extensively replaced with m1Ψ [21, 22]. This nucleoside modification can introduce local structural perturbations that promote ribosomal slippage during translation, potentially resulting in +1 frameshifting and synthesis of unintended peptide products [23–27].

Since this substitution is repeated often, it increases the risk of modRNA mistranslation, potentially eliciting unintended immune reactions. While the frequency and clinical relevance of such events remain debated, their existence challenges earlier assumptions of translational fidelity [25, 28, 29].

Although early industry‐sponsored studies claimed that there was no mistranslation from modRNA [30], more recent mass‐spectrometry analyses of BNT162b2‐vaccinated individuals identified frameshift‐derived peptides that elicit cellular immune responses [21, 25, 29]. Independent immunopeptidomics studies have also documented T cell recognition of +1 frameshift‐derived products following vaccination [25].

Perhaps of greater concern is also that the postribosomal metabolic pathway of m1Ψ is unclear, leaving uncertainty regarding its degradation, excretion, or potential reincorporation into cellular RNA or DNA. Data on specific degradation enzymes, excretion pathways and the possibility of reincorporation into cellular RNA or DNA at supraphysiological concentrations are limited. Some researchers have pointed out that individuals experiencing new‐onset adverse events or cancer after modRNA vaccination exhibit widespread transcriptomic dysregulation [31].

#### 3.1.2. Problem 2: Quality Control of modRNA Integrity

Adding to these risks, leaked European Medicines Agency (EMA) documents revealed significant differences in RNA integrity between clinical and early commercial batches (dropping from ∼78% to ∼55% intact modRNA), with truncated species whose safety and efficacy implications were initially undefined [32, 33]. This underscores potential for incomplete or aberrant translation products, disrupting the smooth operation of cellular machinery and raising questions about why such findings were published without greater scrutiny.

#### 3.1.3. Problem 3: Biodistribution and Off‐Target Transfection by LNPs

Preclinical biodistribution studies in rats using luciferase‐encoding LNPs (submitted to regulators) showed that the LNPs, which are vectors for the modRNA vaccines, do not remain at the injection site. Instead, significant accumulation occurs in the liver, spleen, adrenal glands, ovaries and bone marrow within 24 h [14]. Any cell absorbing these LNPs and producing spike protein could turn into a target for the immune system, potentially leading to inflammation or even autoimmunity, disorders that could silently build in the population over time. A study into the persistence of vaccine artifacts in bodily fluids was recently discussed in a systemic review by Selem et al. [34]**.**

There are also secondary routes with which the modRNA might circulate around the body. Cells transfected with modRNA can package the RNA into extracellular vesicles, which can be released and taken up by distant cells, leading to secondary protein production far from the injection site [35]. These vesicles have been reported to cross the blood–brain barrier, travel along nerve fibres, and potentially reach germline cells [36].

This mechanism is reinforced by evidence that BNT162b2 vaccination induces circulating extracellular vesicles expressing SARS‐CoV‐2 spike protein as early as day 14 post first dose, prior to detectable antibodies, suggesting a novel immune activation pathway [12].

#### 3.1.4. Problem 4: Challenges of LNPs

LNPs are crucial for modRNA vaccine delivery, as they protect the payload and facilitate cellular uptake; thus, their effects are a key component of the overall product data in current formulations. However, reviews published since 2021 continue to emphasise that LNPs are not immunologically inert, but actively stimulate innate immune pathways, including Toll‐like receptor signalling and inflammasome activation, with consequences for reactogenicity and systemic inflammatory responses [37, 38]. Experimental work has demonstrated that ionisable lipid components of clinically used LNPs can activate NF‐κB and IRF pathways even in the absence of mRNA cargo, indicating that innate immune activation is an intrinsic property of the delivery vehicle itself [39].

For instance, in 2016, an LNP RNA‐based drug for a rare cardiac condition was withdrawn after there were 6% more deaths in the treatment group [40]. After decades of workup in cancer treatments and gene therapy, the first gene therapy using RNA delivered by LNPs was only approved by the FDA in 2018, i.e., just 2 years before the development of COVID‐19 vaccines [7].

A growing body of evidence suggests that nanoparticles can adversely affect the reproductive system [41]; for example, LNPs have demonstrated accumulation in ovarian tissue [42], whereas cationic liposomes used in gene delivery have been linked to additional immunological and toxicological concerns [43]. Indeed, repeated administration of PEGylated nanoparticle systems, including lipid‐based nanocarriers, has been associated with complement activation‐related hypersensitivity reactions and anti‐PEG antibody formation, which can alter pharmacokinetics and increase immune‐mediated clearance upon subsequent dosing [44–46]. Additional safety concerns include hepatic accumulation, inflammatory and innate immune activation, and, under certain experimental conditions, direct cytotoxic effects observed in vitro [47].

In the context of advanced LNP development, these findings highlight great opportunities for optimisation, such as redesigning ionisable lipids to improve biodegradability and tolerability. Newer formulations incorporating ester‐linked or ketal‐ester ionisable lipids have shown reduced hepatotoxicity, altered organ tropism, and improved safety profiles in preclinical models when compared with earlier lipids used in mRNA vaccines such as SM‐102 and ALC‐0315 [38, 48]. Parallel work has sought to address the well‐described tendency of conventional LNPs to accumulate in the liver by modifying lipid composition and removing structural elements such as cholesterol that contribute to hepatic uptake [49]. Taken together, these developments indicate that the LNP formulations deployed during the initial large‐scale rollout of mRNA vaccines represented an earlier stage of platform development, with recognised biological activity and tolerability constraints that are now the subject of ongoing optimisation. Future advancements in LNPs could mitigate these risks while maintaining their essential role in modRNA delivery.

Further research has elucidated mechanisms underlying LNP‐associated inflammation. A study from University of Pennsylvania researchers, including Nobel Prize winner Dr. Drew Weissman, demonstrated that modRNA‐LNPs exacerbate pre‐existing inflammation (IE) in models of acute inflammation, with increases in cytokines such as IL‐6 up to 14‐fold and MIP‐2 up to 11‐fold compared to inflammation alone [50]. The study, published in December 2021, identified this IE phenomenon as specific to the LNP and independent of the mRNA cargo, and stated that mitigation strategies for IE are warranted to prevent adverse reactions in patients with pre‐existing inflammatory conditions, such as those with gram‐negative bacterial infections [50].

Building on this, a 2024 preprint by Weissman and colleagues revealed that LNPs trigger inflammation through endosomal membrane damage during escape, which creates large, irreparable holes sensed by cytosolic galectins, leading to downstream inflammatory responses [51]. The authors claim that while LNP‐based mRNA vaccines are successful in their estimation, their highly inflammatory nature significantly worsens safety and represents a major hurdle to be overcome in relation to broader LNP therapeutics. In this case they propose re‐engineering with rapidly biodegradable ionisable lipids that create smaller, reparable endosomal holes via the ESCRT pathway or using galectin inhibitors to reduce inflammation, enabling safer use in patients with inflammatory diseases, including the elderly. With such modifications, the authors estimate that LNPs may dramatically improve in these populations [51]. These findings underscore that challenges remain for expanding the modRNA platform beyond vaccines.

#### 3.1.5. Problem 5: Choice of Spike Protein as an Antigen

The persistence and biological activity of the SARS‐CoV‐2 spike protein have led some researchers to propose unifying hypotheses to explain clusters of postvaccination symptom reports. While terminology such as “postvaccination syndrome” has been used descriptively in this context, these constructs are not yet formally defined clinical entities and remain the subject of ongoing investigation. Nevertheless, several studies have challenged the assumption that the COVID‐19 spike protein is uniformly short‐lived and biologically benign [14, 52].

In addition to this:

• It has been reported that circulating spike protein can be found associated with monocytes for as long as 245 days [53] In addition, some preprint studies have reported detection of the vaccine‐derived S1 protein fragment in participants′ plasma for as long as 709 days [54].
• Spike protein can persist in the cerebral artery intima in 43.8% of vaccinated patients up to 17 months postvaccination [55].
• Experimental studies have reported that spike protein may promote the formation of in vitro amyloid‐like fibrils, with potential implications for clotting and neurological pathology [56].
• Spike protein and its coding RNA have been observed inside the cell nucleus, even in natural SARS‐CoV‐2 infections [57], raising questions regarding possible parallels with vaccine‐derived spike protein persistence. Binding of spike protein to ACE2 receptors has also been reported to disrupt the renin–angiotensin system (RAS). Dysregulation of this pathway has been implicated in severe COVID‐19 and has also been proposed as a potential mechanism underlying certain adverse events following spike protein‐based vaccination [58, 59].
• Some authors have proposed that spike protein exposure could indirectly contribute to oncogenic processes through mechanisms involving p53 pathway interference, chronic inflammation and immune dysregulation within a multihit, nongenotoxic framework [60–64].

#### 3.1.6. Problem 6: Plasmid DNA Contamination and the Risk of Core Genomic Integration

Mass production of the new type of vaccine relies on plasmid templates grown in *E. coli*. Several laboratories have now reported residual plasmid DNA (including SV40 promoter/enhancer sequences in some Pfizer lots) at levels 36–627 times above regulatory limits [65]. Further methodological critiques emphasise that qPCR underestimates DNA impurities in Comirnaty by 360–534 times compared to fluorometry, underscoring the inadequacy of current quantification methods and heightening risks of genomic integration [66]. Independent analyses of BNT162b2 lots confirm residual DNA at 32.7–43.4 ng/dose, including SV40 promoter/enhancer sequences, exceeding limits and enabling cellular uptake with potential integration risks [65, 67].

One reported case has raised the possibility of apparent genomic integration of vaccine‐derived sequences associated with aggressive cancer [68], although this observation requires independent confirmation.

This contamination poses heightened dangers, especially during mitosis, when the nuclear envelope breaks down. This temporarily removes genomic protection in dividing cells within proliferative tissues, allowing foreign DNA easier access to the genome. As historical research demonstrates, a single mutated oncogene (such as the Ha‐ras‐1 gene from the T24 bladder carcinoma cell line) can induce complete malignant transformation of early passage cells without requiring a cooperating gene, suggesting oncogenes like those promoted by SV40 sequences may act at earlier stages of tumorigenesis than previously suspected [69]. The extent of this SV40 contamination is still a highly debated topic [67], but if high levels are found, it could lead to further oncogenic risks.

#### 3.1.7. Problem 7: Innate Immune Dysregulation From Foreign Nucleic Acids

The risk to innate intracytoplasmic defences against foreign DNA/RNA from the modRNA manufacture process appears significant and potentially harmful. Lab experiments indicate that cytoplasmic nucleic acids can activate innate immune defences via pathways such as cGAS–STING, which detect aberrantly localised DNA and initiate downstream inflammatory and interferon responses [70]. The relationship between foreign DNA/RNA and activation of the immune system cascades has been linked to epigenetic changes [71] and has been proposed as one of several mechanisms that may contribute to adverse events and cancer following vaccination [31, 72, 73]. While these responses are normally controlled, large boluses of foreign nucleic acids may have the potential to dysregulate cellular processes and represent one possible mechanism contributing to pathologies that have been reported following modRNA and viral vector vaccination:

Type 1: Transcriptomic dysregulation (thousands of genes affected, ribosomal disruption and mitochondrial collapse) [31].

Type 2: Proteomic abnormalities (aberrant folding, changes in fibrinogen/actin/amyloid proteins and seeding clotting) [56, 74].

Type 3: Systemic adverse events, including myocarditis, clotting, fatigue, immune disorders and death, which have been reported following vaccination [31, 75, 76].

In addition, manufacturers generally maintain that synthetic foreign DNA/RNA has minimal impact on host factors. Nevertheless, theoretical or proposed risks include overstimulation of intracellular defence pathways, epigenetic alterations, chromosomal integration and carcinogenesis, particularly in the context of respiratory RNA viruses. These issues warrant further safety studies that are independently reproducible outside contracted laboratories [31].

#### 3.1.8. Problem 8: Endotoxin Contamination

The use of *E. coli* in plasmid production is standard in modRNA vaccine manufacturing but it also carries risks such as endotoxins that can provoke systemic inflammatory responses, including anaphylaxis, if not rigorously removed. Indeed, BioNTech acknowledged in a peer‐reviewed publication that vaccine manufacturing by‐products may remain despite purification and can trigger innate immune responses [77].

During modRNA vaccine development, two distinct formulations of the Pfizer/BioNTech vaccine were used. The original formulation, process 1 (P1), was deployed in pivotal Phase 2/3 trials and early rollout, while a second formulation process (P2) was introduced in late 2020 to enable large‐scale manufacturing under emergency use authorisation (EUA). P2 involved substantial process changes compared with P1, including a switch from PCR‐derived to linearised plasmid DNA templates, larger production scale, modified purification steps, adjustments in nucleotide volumes and incorporation of the ionisable lipid ALC‐0315 [32, 78]. These changes affected RNA integrity, poly(A)tail distribution, protein expression profiles and key physicochemical properties of the LNP formulation, including particle size, polydispersity, zeta potential and colloidal stability [32, 33, 65]. Although P1 and P2 were not distinguished for regulators or clinicians, internal documentation indicates differences in impurity profiles, including residual plasmid DNA and potential endotoxin burden [32, 65]. Most trial participants received P1, while later recipients, during crossover and postlicensure phases, received P2.

These different formulation parameters are critical to biodistribution, cellular uptake, and immunogenicity. Consequently, process‐scale modifications introduced the possibility of altered batch‐to‐batch consistency and increased reactogenicity. Regulatory summaries indeed reported higher rates of certain adverse events, including lymphadenopathy, following the rollout of P2 compared with P1. These observations underscore the importance of rigorous control of endotoxin levels, LNP physicochemical characteristics, and in‐use stability to ensure reproducibility and safety [78, 79].

Early postdeployment surveillance identified several cases of anaphylaxis among initial vaccine recipients, including cases reported in the first days of rollout [80–82]. By contrast, the P1 trial reported no cases of anaphylaxis among approximately 20,000 participants following the first two doses [83]. A single later case involved an adolescent who had received placebo doses followed by one active dose, likely P2, which triggered anaphylaxis [84].

By March 2021, lymphadenopathy had emerged as a frequent postvaccination finding. In breast cancer screening cohorts, rates reached 11% after dose one and 16% after dose two, far exceeding the background rate of less than 0.05% [85–87]. In contrast, the P1 trial reported a rate of 0.4%, classified as uncommon [88]. Substantially higher rates were observed with P2: 4.3% in adolescents and 5.2% in adults receiving booster doses, representing more than a 13‐fold increase relative to P1 [89]. Although Pfizer attributed the higher rate to booster dosing, elevated incidence was evident following initial P2 doses and was not confined to boosting. Notably, 85% of cases occurred after the first or second dose, with some persisting beyond 6 months [90].

Additional trial data indicate that placebo recipients vaccinated later (December 2020) and thus likely receiving P2 reported adverse events at approximately twice the rate of those originally given P1, effectively eliminating long‐term placebo comparison. Pfizer characterised this increase as “expected” [91].

While lymphadenopathy is often considered a minor adverse event, it is clinically meaningful as a marker of systemic immune activation and, in some cases, persistent inflammation. Whether this heightened response reflects endotoxin contamination, residual plasmid DNA, altered LNP properties, or a combination of factors remains uncertain. Nevertheless, postauthorisation surveillance and subsequent clinical studies identified higher rates of lymphadenopathy and other reactogenicity signals than were reported in the original pivotal trials [80, 87, 89, 90]. These observations highlight the importance of continued evaluation of manufacturing consistency, formulation characteristics and residual impurities as potential contributors to vaccine reactogenicity. Given the limited duration of the initial placebo‐controlled follow‐up, continued investigation of the mechanisms and longer‐term implications of these findings remains warranted.

#### 3.1.9. Problem 9: Risk of Endothelial Injury

Intramuscular injection of modRNA vaccine formulations can result in unintended systemic exposure due to inadvertent intravascular administration, estimated to occur in approximately 1.5%–2% of cases even when aspiration is performed [92–94]. During mass vaccination campaigns, however, needle aspiration was widely discouraged, and injections were often administered outside the optimal deltoid “safe triangle,” increasing the likelihood of vascular entry [95].

Historically, standard immunisation training has emphasised aspiration to confirm that the needle is not positioned within a blood vessel, thereby reducing the risk of intravenous delivery. For modRNA‐LNP formulations, however, this practice introduces an additional technical consideration: LNPs are sensitive to mechanical stress, and syringe manipulation during aspiration may disrupt particle integrity, alter colloidal stability, or modify release kinetics [78, 96]. This susceptibility to shear‐related effects likely contributed to the shift away from routine aspiration during large‐scale deployment. A consequence of this policy change may have been an increased probability of inadvertent systemic or endothelial exposure in a subset of injections.

Preclinical studies support the relevance of administration route, demonstrating markedly different biological effects following intravenous versus intramuscular delivery of modRNA–LNP vaccines [97], although direct extrapolation to humans remains uncertain. Complementing this, Moderna reported that only a small fraction (approximately 1%–2%) of administered LNPs successfully enter target cells under experimental conditions, implying that a substantial proportion may remain extracellular or interact with nontarget tissues [98, 99]. Girardot [92] further hypothesises that vascular endothelium may be exposed to comparatively high concentrations of circulating LNPs, particularly in capillary‐dense organs.

Consistent with this hypothesis, autopsy studies have documented findings such as vasculitis and endothelial damage in selected cases of postvaccination death [97, 100, 101]. Disruption of the endothelial barrier can lead to downstream effects including vascular leakage, microthrombosis and tissue‐specific inflammatory responses. This endothelial‐injury pathway represents one of several mechanisms that may contribute to reported cases of myocarditis, stroke and multiorgan inflammatory syndromes following modRNA vaccination, alongside other immune‐mediated and inflammatory processes that remain under investigation.

In response to concerns surrounding aspiration and injection accuracy, alternative device‐based approaches have been proposed. One such concept is a dual‐chamber syringe designed to distinguish between interstitial and intravascular needle placement without requiring plunger retraction, thereby reducing mechanical stress on LNP formulations while preserving injection safety [102]. Such innovations highlight the technical challenges associated with balancing formulation stability and precise delivery in mass vaccination settings.

### 3.2. Preclinical Risk Assessment

A key question in clinical risk assessment is whether these potential issues with the new genetic vaccine technology were acknowledged during EUA (Table 1)? Although manufacturers claim strict purification processes to ensure DNA contaminants remain below levels that trigger adverse effects or unintended immune responses, some independent reports indicate plasmid DNA levels were above recommended thresholds, potentially activating innate immune pathways [31, 33, 65, 72, 103, 104].

**TABLE 1:** What was actually known at time of EUA (2020–2021) vs. what was actually poorly characterised or unknown.

Actually known at time of EUA (2020–2021)	Actually poorly characterised or unknown
Short‐term safety in small trials	Long‐term safety of the platform

Immunogenicity (not protection against infection or disease) in healthy adults	Effects of repeated booster doses

Sequence optimisation (codon, humanisation)	Full effects of extensive m1Ψ substitution

LNP delivery to muscle cells	Whole‐body biodistribution and exosome spread

Duration of spike production‐long‐term persistence had not been studied	Persistence of spike protein for months–years
	Frameshifting and off‐target protein production
	Risk and consequences of plasmid DNA contamination and genomic integration

This disparity between presumed and actual understanding of risk echoes a pivotal lesson from toxicology history. The thalidomide tragedy of the late 1950s–early 1960s arose because the drug consisted of two almost identical mirror‐image molecules: the R‐enantiomer, a safe sedative, and the S‐enantiomer, a potent teratogen. This single atomic rearrangement caused tens of thousands of birth defects and directly led to modern rigorous toxicology requirements [105].

Therefore, modern risk assessment for new pharmaceuticals extends beyond mere academic or regulatory formalities; it represents a framework forged from hard‐learned lessons in toxicology, often drawn from unfortunate historical incidents that underscored the need for evolving safety standards [106]. As such, when policymakers invoke emergencies to propose shortcuts, they must proceed with the utmost caution, recognising that current processes are already streamlined to their practical limits. Moreover, these tests are thoughtfully designed with the end goal of safe commercial products in mind, incorporating the legacy of past expediency to prevent avoidable harms. Therefore, even seemingly minor nucleoside sequence or formulation changes in modRNA vaccines, such as codon optimisation, m1Ψ substitution, LNP composition, or residual contaminants, warrant similar rigorous scrutiny.

It should also be noted that mandatory controls for gene therapy products (GPTs) were by‐passed in the case of modRNA vaccine technology since they were classified as a vaccine rather than a GTP [107].

A clear example of this abbreviated approach is evident in the timelines for BNT162b2 vaccine development. Animal studies commenced on March 17, 2020, (Study No. 38166) using variant 8 (V8: full‐length SARS‐CoV‐2 spike glycoprotein + two proline substitutions) [108, 109], concluding around April 23, 2020 (terminal euthanasia on day 38); full results (clinical pathology, serology and histopathology) were unavailable until August 12, 2020 [109, 110]. Subsequent animal studies included further toxicity testing (Study No. 20GR142, starting July 6, 2020) and reproductive/developmental toxicity (starting July 27, 2020), both with Variant 9 (V9) [109]. Preclinical data from mouse and nonhuman primate models, mainly using V9, were announced September 9, 2020 [111, 112]. Both V8 and V9 showed similar safety profiles, including immune‐related effects like injection site inflammation and reversible liver changes; differences were partly due to dosing (100 μg for V8 vs. 30 μg for V9) [108, 109]. However, while V8 and V9 theoretically encode the same protein, V9 is codon‐optimised, increasing cytosine and reducing uridine, yielding a different nucleotide sequence with stronger immune responses; V9 was introduced later (e.g., July 2020 studies) and became the basis for most preclinical data in mice/nonhuman primates, as well as the final Phase 3 vaccine [108, 112]. In addition codon optimisation is not biologically neutral: synonymous changes alter translation kinetics and co‐translational folding, causing differences in conformation, stability, and function despite identical amino acids [113, 114]. Thus, deploying codon‐optimised modRNA in humans before full animal testing assumes unproven equivalence, creating a nontrivial gap in preclinical risk assessment.

Human trials (Phases 1/2/3, NCT04368728) began April 29, 2020, after Study No. 38166’s in‐life phase but before its full results or V9 animal studies [115]. Phase 3 ran from July 27, 2020, to February 10, 2023 [115, 116]. This accelerated timeline advanced human testing on preliminary V8 data, while V9‐specific studies followed without retroactive impact on Phase 3.

In the case of the Pfizer/BioNTech vaccine some might argue that given the emergency of the COVID‐19 pandemic [117], it might have been justified to allow new technologies to be placed on the market without the standard preclinical safety tests. However, it was evident early on that the conditions for a minimalist preclinical safety strategy were not met, as COVID‐19 was found to not be comparable to very dangerous viruses such as Ebola which is 100 to 1000 times more deadly than COVID‐19 [15].

If Pfizer/BioNTech wished to pursue approval with their abbreviated toxicity programme in order to get to market in a timely manner, we suggest that the following facts should have been considered:

1. Despite 20 years of research, no comparable product based on the mRNA technology platform has been successfully approved.
2. The efficacy of the vaccines is difficult to prove, and the risk of vaccine damage is unknown.
3. According to the Nuremberg Code, people who do not consent to vaccination cannot be coerced, and work‐related vaccination requirements cannot be imposed.
4. If all persons, including children, adolescents and pregnant women, were to be vaccinated, then all internationally required preclinical safety tests, including those for galenic excipients, would have had to be carried out prior to approval.

Attempts were made to include pregnant women in clinical evaluation; however, no dedicated trials in this population were successfully completed [118]. This absence highlights a broader limitation in trial design and population coverage. In parallel, fundamental methodological issues remained: in a study intended to evaluate efficacy against an infectious disease, it is widely regarded as unacceptable not to systematically test all participants for infection status. Together, these gaps underscore the incomplete nature of both safety and efficacy assessment at the time of rollout.

Under these circumstances, in retrospect, we argue that the health authorities should have carefully considered the incomplete preclinical safety dossier submitted by Pfizer and BioNTech. However, despite these considerations, most health authorities granted EUA for the modRNA vaccine technology. The early modRNA vaccine trials, like Pfizer’s, were, on the face of it, presented as highly successful. However, we argue that there were still remaining concerns over how the safety and efficacy data were interpreted [16].

#### 3.2.1. The Clinical Trial Data

As discussed in Quinn et al. [16], although the trials reported relative risk reductions of 66%–95% for symptomatic COVID‐19, the corresponding absolute risk reductions were only about 1%. This distinction is important for contextualising expected population‐level benefit, particularly when evaluating interventions intended for widespread use across heterogeneous risk groups (Table 2).

**TABLE 2:** Summary of the first COVID‐19 genetic vaccine trial data, relative risk reduction (RRR) and absolute risk reduction (ARR).

Vaccine	Participants		Cases identified		RRR %	ARR %	Vaccine type
	Vaccine	Control	Vaccine	Control			
Pfizer/BioNTech	21,720	21,728	8	162	95.06	0.71	modRNA/LNP
Moderna	15,210	15,210	11	185	94.05	1.14	modRNA/LNP
Oxford AZ	5807	5829	30	101	70.18	1.22	DNA viral/vector
Johnson and Johnson	19,630	19,691	116	348	66.56	1.18	DNA viral/vector
Sputnik	16,501	5476	16	62	91.41	1.04	DNA viral/vector

*Note:* Modified RNA + Lipid Nanoparticle (LNP) vaccines (modRNA/LNP), Adenoviral vector (DNA‐based delivery) vaccines (DNA viral/vector). Adapted from “What Lessons can Be Learned From the Management of the COVID‐19 Pandemic?” [16].

For example, in Pfizer’s clinical trial that included 43,448 participants, the authors reported a 95% relative risk reduction in symptomatic COVID‐19 (162 cases in the placebo group versus 8 in the vaccinated group). Yet the absolute risk reduction was only 0.7%, a very marginal gain. It should also be noted that the trials were not powered to assess protection against severe disease or death; absolute risk reductions for these outcomes were modest [119, 120].

#### 3.2.2. Suspected but Unconfirmed COVID‐19 Cases Ignored

The research trial data included an additional underreported category known as “suspected but unconfirmed COVID‐19” which was not considered in RRR calculations. In the Pfizer trial results there were 1594 cases of suspected but unconfirmed COVID‐19 in the vaccine group and 1816 in the placebo group. These numbers were 20× higher (3410 total) than the 170 confirmed cases used for calculating RRR. Scientist Peter Doshi noted these cases were apparently not tested by RT‐PCR, so they were excluded from the results, potentially skewing the assessment of risk while also being a missed opportunity to assess more safety data [121]. It is still unclear how many of the 3410 suspected but unconfirmed cases in the Pfizer trial would have been confirmed cases if adequately tested and how this would have affected the RRR. It was also noted that a further 371 participants (311 from the vaccine group vs. 60 from the placebo group) were excluded from final analysis for “important protocol deviations on or prior to 7 days after Dose 2” [121].

It is also notable that there was not one COVID‐19‐related death in the trial of 43,448 participants. Indeed, total deaths (from any cause) after the follow‐up period were 15 in the vaccinated group and 14 in the placebo group [122, 123]. There was no net mortality advantage and therefore no statistical justification for deviating from the null result. However, as we have stated, clinical trials were not designed to assess protection against severe disease or death [119, 120].

Another issue that many policy makers and members of the public might have missed is the low infection fatality rate apparent from trial results. We remind the reader that the original reported benefit of the vaccine was that it would reduce rates of symptomatic COVID‐19 with an RRR of 95%. The potential influence on severe COVID‐19 could not be evaluated during the clinical trial due to the low incidence in either arm.

#### 3.2.3. Trial Irregularities and Whistle–Blower Testimony

In November 2021, a former regional director of one of the Pfizer clinical trial sites, Brook Jackson, provided whistle–blower testimony alleging serious concerns over data integrity and regulatory oversight in the site she inspected [124]. This included undocumented participant unblinding (where trial staff could potentially identify who received the vaccine or placebo) and evidence that complaints from some trial participants were not being adequately followed‐up or even recorded [124]. Both Brianne Dressen (AstraZeneca trial participant) and Maddie de Garay (Pfizer 12–15‐year‐old trial participant) publicly spoke about how:

a. Their severe adverse reactions were omitted from the papers describing the trial results;
b. They experienced psychological manipulation (“medical gaslighting”) rather than medical support from organisers of the trials that they participated in;
c. They were still struggling with severe vaccine injuries years after the trials [125, 126].

### 3.3. Statistical Interpretations of Early Observational Risk Assessments

Despite these concerns about the safety and efficacy of COVID‐19 vaccines, by mid‐2021 many observational studies seemed to verify that the effectiveness of the genetic vaccine was more than 90% in the short time frame [127, 128]. However, most of these observational studies have been contested by independent statisticians who claimed that they could be subject to statistical biases [123, 129–134].

One of the most frequently cited papers used to promote the safety and effectiveness of COVID‐19 genetic vaccines was the model‐based analysis by Watson [128]. The authors estimated that 14.4 million lives were saved during the first year of vaccination across 185 countries by comparing observed mortality data with a counterfactual scenario in which no vaccines were available.

In this counterfactual framework, the authors modelled the number of deaths expected in the absence of vaccination and attributed the difference between these projections and actual observed deaths to vaccine deployment. This approach implicitly assumes that any deviations from the modelled mortality trajectory are primarily attributable to vaccination, rather than to other contemporaneous factors [130].

The principal limitation of this reasoning is that it treats the vaccine as the dominant explanatory variable while inadequately accounting for multiple confounders known to influence COVID‐19 mortality trends. These include seasonality, accumulated infection‐derived immunity, demographic heterogeneity in risk, changes in testing and reporting, attenuation and evolution of the virus, changes in treatment protocol and the availability of repurposed early treatments such as ivermectin, as well as substantial alterations in human behaviour over time [135–137].

Systematic reviews and meta‐analyses as early as 2021 reported potential reductions in mortality and disease severity with ivermectin [138, 139], while large‐scale implementation of ivermectin‐based protocols in regions such as Uttar Pradesh and Goa, India, coincided with marked declines in COVID‐19 cases and hospitalisations [140]. In addition, the Watson et al. model assumes a consistently high vaccine effectiveness throughout the year, despite evidence of waning protection and variant‐dependent differences in effectiveness [123, 132, 133].

Subsequent critiques have highlighted multiple problematic assumptions underlying the Watson et al. assumptions, including (i) a persistently high vaccine effectiveness across time and populations; (ii) stable and uniform levels of viral circulation; (iii) homogeneous population susceptibility; and (iv) the absence, or minimal impact, of naturally acquired immunity [123, 133, 141].

Watson et al.’s assumptions are also difficult to reconcile with real‐world epidemiological data. For example, approximately four million COVID‐19 deaths were recorded globally in the year preceding vaccine availability, when the virus was more virulent and population immunity was minimal, compared with roughly three million deaths in the subsequent year despite widespread viral circulation and ongoing pandemic dynamics [133]. Moreover, it was already well established that COVID‐19 mortality risk was highly age‐stratified, with older adults bearing the vast majority of deaths, while adolescents and younger populations faced orders‐of‐magnitude lower risk [16, 123, 128, 133, 142]. Seasonal patterns in infection and mortality were also documented across multiple regions prior to and during vaccine rollout [135, 143, 144] further challenging the validity of a static counterfactual baseline.

#### 3.3.1. Types of Statistical Bias in Observational Studies

Analyses have identified potential statistical biases in several COVID‐19 vaccine observational studies. These bias include miscategorisation bias, age bias, background infection rate bias, healthy vaccine effect and counterfactual scenarios [130, 132, 133, 145, 146]. Methodological critiques have argued that some studies may be susceptible to exposure misclassification when vaccinated status is assigned only after a specified postvaccination interval, thereby creating periods during which outcomes are attributed to the unvaccinated group or excluded from analysis [132, 145]. The potential impact of such design choices depends on the study design, outcome definition and analytical approach employed. More broadly, observational vaccine studies remain vulnerable to confounding and selection effects that may influence effect estimates if not adequately addressed through study design and statistical adjustment.

#### 3.3.2. The Gradual Evolution of the Reporting of Adverse Events

When discussing adverse reactions associated with drugs (pharmacovigilance), the terms “very rare”, “rare” and etc. are often used (Table 3).

**TABLE 3:** Common notation used in pharmacovigilance.

Term	Frequency (ratio)	Percentage (%)	Per million patients
Very Common	≥ 1 in 10	≥ 10%	≥ 100,000
Common	≥ 1 in 100 and < 1 in 10	≥ 1% to < 10%	≥ 10,000 and < 100,000
Uncommon	≥ 1 in 1000 and < 1 in 100	≥ 0.1% to < 1%	≥ 1000 and < 10,000
Rare	≥ 1 in 10,000 and < 1 in 1000	≥ 0.01% to < 0.1%	≥ 100 and < 1000
Very Rare	< 1 in 10,000	< 0.01%	< 100

*Note:* Adapted from European Medicines Agency [147].

Data on side effects of modRNA vaccines emerged very slowly in scientific reports and peer reviewed publications after the roll‐out of the modRNA vaccines.

#### 3.3.3. Adverse Events Involving Myocarditis/Pericarditis

Early reports of adverse events such as myocarditis and pericarditis after vaccination were initially described as “very rare” by official and pharmaceutical sources [148]. In initial studies, Pfizer reported myocarditis at a rate of 2.7 per 100,000 for vaccines vs. 11 per 100,000 for COVID‐19 infection (i.e., claiming there was a greater risk from COVID‐19 infection than COVID‐19 vaccination) [149]. It was later realised these comparisons were flawed because rates of myocarditis following infection were much rarer since in the early stages of COVID‐19 testing infection rates had been substantially underestimated.

As more comprehensive data emerged, a more complex and less consistent picture became apparent, particularly when stratified by age and sex. Several population‐based studies have reported differing patterns of myocarditis and pericarditis risk following vaccination versus infection. For example, data from the United Kingdom indicate that myocarditis in children and adolescents was documented following vaccination but not observed following infection in the same populations [150]. Similarly, a large Nordic cohort study (Denmark, Sweden, Norway and Finland) found that in individuals aged 16–24, the risk of myocarditis and pericarditis was higher following Pfizer vaccination than after COVID‐19 infection [151]. Similarly, a population‐based Israeli study reported no evidence of increased risk of myocarditis or pericarditis among unvaccinated adults recovering from COVID‐19 [152].

Other observations seem to agree with these assessments, for example:

1. Le Vu et al. found a 1 in 10,000 rate of myocarditis/pericarditis in vaccinated people under 50%, and 97% of cases could be directly attributed to the vaccine. That classified it as “rare” rather than “very rare” [75].
2. Fraiman et al. reanalysed Pfizer and Moderna trial data and found an excess risk of ‘serious adverse events of special interest’ of 10–15 per 10,000 vaccinated (roughly 1 in 800 which is “uncommon”) [17].
3. Mansanguan et al. in Thailand, 13–18‐year‐olds: 29% experienced some cardiovascular manifestation (“very common”) after the 2nd Pfizer dose; 3.5% of boys developed subclinical myopericarditis (all reportedly recovered within 2 weeks) [153].

Although some of these adverse reactions have been described as ‘mild and transient’, the heart has a very limited capacity of repair following significant injury [154–157]. Indeed, follow‐up studies after modRNA COVID‐19 vaccines have shown that despite inflammatory and cardiac biomarkers returning to normal ranges, one‐third of patients who had been admitted for clinical myocarditis continue to experience symptoms [76].

In that context, it has to be noted that myocarditis is clinically challenging to diagnose due to its heterogeneous and often nonspecific presentation, with symptoms and diagnostic findings that frequently overlap with other cardiac conditions such as acute coronary syndromes [158, 159]. Furthermore, the presence of asymptomatic and subclinical cases, together with limitations in diagnostic sensitivity, suggests that the true incidence of myocarditis may be underestimated, with hospital‐diagnosed cases likely representing only a subset of affected individuals [159].

Indeed, there is strong evidence of subclinical (silent) heart damage, especially in teenage boys (where ∼29% had symptoms and ∼3% had elevated troponin levels) [153]. The long‐term implications of this widespread subclinical damage are concerning, particularly given the missed opportunities for a thorough investigation.

Recent mechanistic studies provided at least some biologically plausible explanations for modRNA vaccine‐associated myocarditis. Cao et al. [58] report that vaccine‐derived foreign mRNA activates innate immune sensors, particularly TLR3, in cardiac macrophages, inducing CXCL10 release and recruitment of IFN‐γ–producing T cells. Rather than direct immune cell–mediated cytotoxicity, cardiomyocyte injury appears to occur through IFN‐γ–driven intracellular stress, with upregulation of immunoproteasomes that degrade sarcomeric proteins, leading to cardiomyocyte self‐destruction. This mechanism would represent a class effect of modRNA technology [103]. Other scientists have proposed that intracellular processing and aggregation of the encoded spike proteins contribute to cardiac cellular stress [160].

#### 3.3.4. Other Adverse Reactions to Vaccines Derived From Genetic Technologies

Over time it is becoming clearer that there are multiple other adverse outcomes from genetic vaccines such as cerebral thrombosis [161], encephalomyelitis [162], cancer [163, 164], psychiatric reactions [165, 166], fertility problems [167, 168], tinnitus [169], neurological side effects [170], shingles [171] and fatal outcomes in some cases [172, 173]. Indeed, larger studies are confirming an increasing trend: Faksova et al. [18] in 99 million vaccinated individuals, found increased risks of heart attack (+286% after 2nd dose), stroke (+240% after 1st dose), coronary artery disease (+244% after 2nd dose), arrhythmia (+199% after 1st dose) etc. [18]. A further large study of 2.9 million U.S. patients showed that acute kidney injury and dialysis rates were significantly higher in vaccinated individuals, and Pfizer (BioNTech) recipients showed an elevated risk of death compared to unvaccinated controls, even after all early postvaccination deaths were excluded from analysis [174].

#### 3.3.5. Adverse Event Reporting

In the U.S. Vaccine Adverse Event Reporting System (VAERS), approximately 91% of all reported vaccine‐associated deaths since 1990 (∼22,803 out of ∼25,000 as of May 2025) have been linked to COVID‐19 vaccines, exceeding the total reported for all other vaccines combined over the same period. Real‐world observational data have also raised questions regarding effectiveness trends, with some studies suggesting that repeated dosing may be associated with increased susceptibility to COVID‐19 infection, as reported in the Cleveland Clinic cohort [175].

Additional observational findings have pointed to potential increases in certain chronic conditions among vaccinated paediatric populations [176]. In parallel, questions remain regarding the use of the modRNA vaccine in pregnancy. While COVID‐19 itself is generally not associated with severe outcomes in pregnant women in the absence of underlying risk factors [177], there remains limited direct evidence supporting the safety of modRNA vaccines in this population. In contrast, concerns have been raised regarding biodistribution of modRNA vaccines. Early reports suggesting transplacental passage of vaccine‐derived modRNA were initially dismissed; however, multiple subsequent studies have documented evidence consistent with this phenomenon [167, 178–180].

At the population level, similar signals have emerged internationally. Analyses of European data have raised questions regarding the contribution of COVID‐19 and its countermeasures to patterns of excess mortality [181]. In addition, several studies have reported higher age‐adjusted non‐COVID‐19 mortality rates among vaccinated individuals in certain cohorts, along with temporal associations between mortality peaks and vaccination periods, findings that warrant further investigation [182–184].

More speculative but nonetheless concerning perspectives on overall harm include estimates attributing a substantial proportion of excess deaths since 2021 to vaccination. For example, Baudin et al. [185] estimate up to 19 million deaths globally, while Foster et al. [186] estimate 18.2 million deaths by the end of 2023, in addition to 27.9 million “missing babies” potentially linked to reproductive effects of modRNA vaccines. Foster et al. [186] is not itself a peer‐reviewed publication but is based on analyses drawing on mortality and birth data from the United Nations World Population Prospects 2024 database [187] and incorporates estimates derived from other sources. In relation to fertility effects, the estimate of “missing babies” is linked to analyses that have subsequently been published [168]. Related discussion of large‐scale societal harms associated with COVID‐19 policy responses has also appeared in the peer‐reviewed economics literature [188]. We recognise that these estimates, derived from excess mortality analyses and demographic modelling, differ markedly from mainstream models, which primarily attribute excess deaths to COVID‐19 infection and indirect pandemic‐related factors [128]. Hence such figures should be interpreted with caution and remain the subject of substantial debate. They also highlight the need for continued independent investigation and transparent evaluation of population‐level outcomes.

A critical factor complicating the interpretation of vaccine safety data is the well‐documented issue of underreporting of adverse events. This phenomenon has been consistently identified across pharmacovigilance systems for decades. Early analyses, including those by Alvarez‐Requejo et al. [189] and Hazell and Shakir [190], demonstrated substantial underascertainment in spontaneous reporting systems. AHRQ‐funded work using electronic health records further suggested that many vaccine adverse events may not be captured through routine passive reporting mechanisms [191]. The extent of underreporting varies substantially according to the nature and severity of the event, but underascertainment remains a well‐recognised limitation of passive surveillance systems.

More recent investigations have reaffirmed these concerns in the context of COVID‐19 vaccine surveillance. Studies examining national datasets [192], network‐based analyses [193] and systemic barriers to reporting [194] all point to persistent gaps in adverse event capture. In addition, critiques of passive surveillance systems such as VAERS have highlighted issues of underreporting, incomplete data and variable data quality [195].

Taken together, these limitations suggest that passive reporting systems may not fully reflect the true incidence of adverse events. This uncertainty complicates risk assessment and reinforces the importance of robust, active surveillance systems and long‐term follow‐up in evaluating vaccine safety at the population level.

### 3.4. Unresolved Problems With modRNA Vaccine Technology

The literature discussed above has identified several unresolved questions and potential concerns regarding the modRNA platform, including:

1. The potential disruption of cell metabolism and immunity [29].
2. Risk of nonspecific cell targeting [167, 196].
3. Accurate measurement of dose and duration of spike protein production [14].
4. The potential inflammatory risk of lipid‐nanoparticles [46], and concerns about their quality control, colloidal stability and dispersion of vaccine preparations [98, 197–199].
5. Potential risk of excretion of modRNA and spike protein [200].
6. Proper risk assessment of the interactions between exosomes, noninfectious viral particles and LNP [12, 52, 201].
7. Potential associations with cancer [62, 163, 164].
8. Reports suggesting the possibility of genomic integration [68].
9. Potential reproductive risks [202].
10. Questions regarding possible platform‐specific myocardial toxicity [103].
11. Uncertainty regarding the long‐term effects of booster‐induced IgG4 subclass switching [175, 203].

## 4. Conclusions

This narrative review has outlined key toxicological and safety concerns associated with the modRNA vaccine platform, spanning molecular design, quality control, delivery and the subsequent biology of the encoded antigen. These issues have been considered alongside emerging evidence of adverse events affecting cardiovascular, neurological, reproductive, and other systems. We acknowledge that preclinical evaluations were abbreviated under emergency timelines, preceding the availability of full variant‐specific toxicology and reproductive data. However, postauthorisation observational studies, although initially reassuring, have been subject to methodological limitations. Subsequent analyses have increasingly identified adverse events previously characterised as “very rare” (e.g., myocarditis/pericarditis, neurological, thrombotic and reproductive effects) at higher‐than‐anticipated frequencies.

A critical aspect of the toxicological risk assessment is that the modRNA constructs and manufacturing processes differed between the pivotal clinical trials and scaled‐up deployment. Early preclinical studies employed Variant 8 (V8), while later animal studies and the final vaccine used codon‐optimised Variant 9 (V9). Although both encode the same amino‐acid sequence, codon optimisation can alter translation kinetics, co‐translational folding, stability and immunogenicity; however, comprehensive bridging studies for toxicological equivalence were not completed before human trials. Similarly, the transition from Process 1 (P1) to Process 2 (P2) involved changes in plasmid templates, purification, lipid composition and RNA integrity, resulting in higher residual DNA, endotoxin burden and increased reactogenicity (> 13‐fold lymphadenopathy and doubled adverse‐event rates). These findings underscore that “process is product” and the absence of adequate comparability data.

Such gaps in comparability and oversight become particularly consequential when regulatory decisions are made under emergency conditions.

Decision‐making under emergency conditions, as seen during the COVID‐19 rollout, may also have influenced how emerging safety signals were interpreted, potentially reinforcing early assumptions before sufficient evidence accumulated [1, 2, 204]. Related economic and institutional analyses suggest that centralised, rapid‐response strategies can reduce emphasis on long‐term safety oversight and constrain open scientific discourse [205–208].

The premature dismissal of dissenting perspectives as “misinformation” and censorship may further limit critical processes such as debate, replication and error correction that are essential to both toxicology and public health [1, 207].

An important limitation of the present review, and of much of the existing literature, is the heavy reliance on cross‐sectional analyses, short‐term pharmacovigilance data, and retrospective observational designs. These approaches provide only snapshots of biological processes that may evolve over years or even decades. While large cohort studies have identified specific adverse events such as myocarditis within narrow risk windows [151], longer‐term follow‐up data still remain concerning [76].

This highlights a broader gap in the toxicological risk assessment of the modRNA platform: insufficient integration of temporal dynamics, predisease processes, and cumulative influences. There is also heterogeneity in individual risk from the platform, including genetic susceptibilities associated with severe outcomes [209] and familial clustering of adverse events [210] which further complicates interpretation. Prospective longitudinal cohort designs incorporating serial biomarker assessment and biobanking, such as the prospective cohort incident‐tumour biobank method (PCIBM) [211] may be important in the characterisation of long‐term platform risks and interindividual variability.

The accelerated deployment of modRNA vaccines should be considered in terms of both the promise and the unresolved risks of this platform. While innovations such as nucleoside modification, codon optimisation, and LNP delivery have addressed key technical barriers, they have also introduced new biological and toxicological complexities, including altered translation dynamics, systemic biodistribution, prolonged antigen expression and potential effects linked to residual plasmid DNA. Although ongoing optimisation, particularly of LNP formulations, may mitigate some of these challenges, the cumulative evidence supports a more cautious and methodologically rigorous approach to future development.

To safeguard public health, further investigation into modRNA vaccine deployment is warranted until these platform‐associated risks are more fully characterised and mitigated. Future applications should prioritise comprehensive preclinical testing, long‐term safety monitoring, and full transparency in data reporting, alongside protection of open scientific discourse, to ensure that technological advancement remains aligned with established toxicological standards and ethical principles.

## Author Contributions

Conceptualisation, Gerry A. Quinn; writing–original draft preparation, Gerry A. Quinn; writing–review and editing, Gerry A. Quinn, Philipp Bagus, Ronan Connolly, Willie Soon, Anton O’Connor, Antonio Sánchez‐Bayón, and Aristidis M. Tsatsakis; methodology and technical consultation on zeta potential, lipid nanoparticle physical characteristics and biochemistry, Daniel Santiago and Anton O’Connor; genomics and oncology expertise, Demetrios A. Spandidos; evaluation of potentially conflicting data, Ricardo F. Savaris; analysis of excess mortality and demographic trends, Paul Frijters; interpretation of innate immunity, adverse event trends and serious adverse events, Steven J. Hatfill; toxicology and regulatory requirements for pharmaceutical approval in pandemic settings, Helmut Sterz and Clare Craig; Michaéla C. Schippers contributed to the conceptual analysis of cognitive and institutional biases, reflexivity and the epistemic conditions for scientific evaluation during crises; clinical trials and epidemiology expertise, Harald Walach.

## Funding

None of the authors received funding from pharmaceutical companies involved in the development or distribution of mRNA products.

## Disclosure

This manuscript was an expanded and fully developed version of material presented in a short oral presentation by Gerry A. Quinn at EUROTOX 2025 (Athens, Greece). No full abstract or proceedings paper was published. All authors have read and agreed to the published version of the manuscript.

## Conflicts of Interest

Gerry A. Quinn, Willie Soon and Ronan Connolly are members of CERES, an independent scientific organisation. Clare Craig and Gerry A. Quinn are members of HART, a voluntary group of doctors and scientists focused on health and recovery. HART has published articles on COVID‐19 mRNA vaccination that evolved with the emerging evidence base. Neither HART nor CERES holds an institutional position for or against mRNA vaccination; both organisations emphasise scientific scrutiny, evidence‐based medicine, and open discussion. Daniel Santiago serves as an unpaid Associate Editor for the International Journal of Vaccine Theory, Practice and Research (IJVTPR). Several co‐authors were signatories of the Great Barrington Declaration (an October 2020 call for evidence‐based discussion of COVID policies in the era before the vaccines were available). Michaéla C. Schippers is the founder of the Great Citizens Movement https://www.greatcitizensmovement.org/. She hosts the public science podcast Follow the Science at https://followthescience.nl/index.html. These activities are unpaid and independent of the present manuscript. Harald Walach is a member and current head of the voluntary group MWGFD in Germany, which informs the public about potential risks of modified mRNA interventions. Paul Frijters is affiliated with the Australian Society for Economists, the Tinbergen Institute, the European Peace Project and Academia Libera Mentis. These affiliations are disclosed in the interest of transparency. The remaining authors declare no conflicts of interest.

## Data Availability

The data supporting the findings of this study are available from the referenced sources listed at the end of the manuscript. No original data were generated or analysed in this review.
